# Intraoperative Triggered Electromyography Recordings from the External Urethral Sphincter Muscles During Spine Surgeries

**DOI:** 10.7759/cureus.4867

**Published:** 2019-06-10

**Authors:** Faisal R Jahangiri, Rabehah A Asdi, Izabela Tarasiewicz, Moutasem Azzubi

**Affiliations:** 1 Neurophysiology, Axis Neuromonitoring, Richardson, USA; 2 Neurology, University of Texas at Dallas, Richardson, USA; 3 Neurosurgery, University of Texas Health Science Center, San Antonio, USA; 4 Neurosurgery, King Abdulaziz Medical City, Ministry of National Guard Health Affairs, Riyadh, SAU

**Keywords:** external urinary sphincters, urinary incontinence, electromyograph, emg, ionm, neuromonitoring, cauda equina tumors, tethered cord, sensory evoked potentials (ssep), motor evoked potentials (mep)

## Abstract

Introduction: Bowel and bladder function are at risk during tumor resection and other surgeries of the conus, cauda equina, and nerve roots. This study demonstrates the ability to acquire triggered electromyography (t-EMG) from the external urethral sphincter (EUS) muscles by utilizing a urethral catheter with an electrode attached.

Methods: A retrospective analysis of neurophysiological monitoring data from two medical centers was performed. Seven intradural tumors and three tethered cord release surgeries that used urethral sphincter electrodes to record t-EMG were included in the analysis. The patients consisted of five females and five males with ages ranging from eight months to 67 years (median: 49 years). Our neuromonitoring paradigm included upper and lower extremity somatosensory evoked potentials (SSEPs) and transcranial electrical motor evoked potentials (TCeMEPs), as well as spontaneous and triggered electromyography (EMG) from the external anal sphincter (EAS), EUS muscles and lower extremity muscles bilaterally. A catheter with urethral electrodes attached was used for recording spontaneous electromyography (s-EMG), t-EMG, and TCeMEPs from the skeletal muscle of the EUS. Train of four (TOF) was also recorded from the abductor hallucis muscle as well for monitoring the level of muscle relaxant.

Results: We were able to successfully record t-EMG responses from the EUS muscles in all patients (100%). It is worthy to note that only one patient presented preoperatively with bladder incontinence, urgency, and frequency. Almost immediately in the postoperative phase, the patient’s frequency and urgency improved, and the bladder function normalized within two weeks of having the tumor removed.

Conclusions: In this small series, we were able to acquire t-EMG in 100% of patients when recorded from the EUS using a urethral catheter with electrodes built into it. T-EMGs can be attempted in surgeries that put the function of the pelvic floor at risk. More study is needed to establish better statistical methods, better modality efficacy, and a better understanding of intraoperative countermeasures that may be employed when an alert is encountered to prevent impending neurological sequelae.

## Introduction

The two urethral sphincters muscles are responsible for controlling micturition and for maintaining urinary continence. The smooth muscle internal urethral sphincter (IUS) is responsible for involuntary control and constriction of the internal urethral orifice. The striated external urethral sphincter (EUS) is responsible for a voluntary control and is part of the somatic nervous system [1]. The pudendal nerve (S2-S3-S4 nerve roots) controls the urethral sphincter. Any damage to the pudendal nerve or EUS may result in a lower urinary tract disorder. This would cause involuntary loss of urine, known as urinary incontinence. Women are more at risk of urinary incontinence because of the anatomy of the pelvic floor musculature and ligaments. During various surgeries, complications involving the urethral sphincter can lead to disorders including urinary incontinence, which can vary in men and women based on anatomical differences.

Bowel and bladder functions are at risk during various surgeries such as spinal surgeries, and tumor resections involving the conus, cauda equina and nerve roots. Multimodality intraoperative neurophysiological monitoring (IONM) is routinely performed during these procedures [2]. Bowel functions are usually recorded only from the external anal sphincter muscles (EAS) and monitored with electromyography (EMG), and transcranial electrical motor evoked potentials (TCeMEPs). Bladder function can now be monitored by TCeMEP from the EUS muscles [3]. Pudendal nerve somatosensory evoked potentials (PSEP) can also be used for monitoring the sensory roots of the pudendal nerve [3]. Somatosensory evoked potentials (SSEP) along with TCeMEP are used for monitoring of the spinal cord function. To monitor the bladder function directly triggered electromyography (t-EMG) can be utilized for intraoperative mapping of the pudendal nerve. This study demonstrates the ability to acquire t-EMG from the EUS muscles by utilizing a urethral catheter with an attached electrode.

T-EMG is an IONM technique that involves electrical stimulation of nerve roots using a hand-held probe, and the measurement of compound muscle action potentials (CMAPs) from muscles innervated by these roots [4]. It is highly useful for surgeries involving the lower abdominal and pelvic areas which put bowel and bladder functions at high risk of neural damage [5]. Utilizing multimodality IONM allows surgeons to monitor the vital functions of the urethral sphincter throughout surgery and avoid causing damage to the surrounding nerves.

Surgeries that take place in the pelvic region have a high probability of causing damage to the bowel and bladder, leading to permanent and potentially fatal postoperative complications. In a study researching the incidence rate of postoperative urinary incontinence following spinal cord tumor surgeries without the use of IONM, it was found that over 83% of patients were left with urinary tract deficiencies [6]. Without IONM assistance and guiding the surgeon throughout the procedure, the likelihood of the patient experiencing bladder dysfunction is significantly increased. If the nerves innervating in the bladder are damaged, other life-threatening complications such as incontinence or urinary retention may persist [7].

With the help of IONM, contemporary technology has allowed surgeons to intraoperatively monitor and test the receptiveness of the urinary sphincter using specialized electrodes to avoid causing lasting damage to the surrounding nerve roots. Triggered EMG recordings have very high specificity and are highly useful in aiding the preservation of autonomic nerves that innervate the bladder and sexual organs, as per the study by Delacroix et al. in 2010 [5].

## Materials and methods

**Figure 1:**
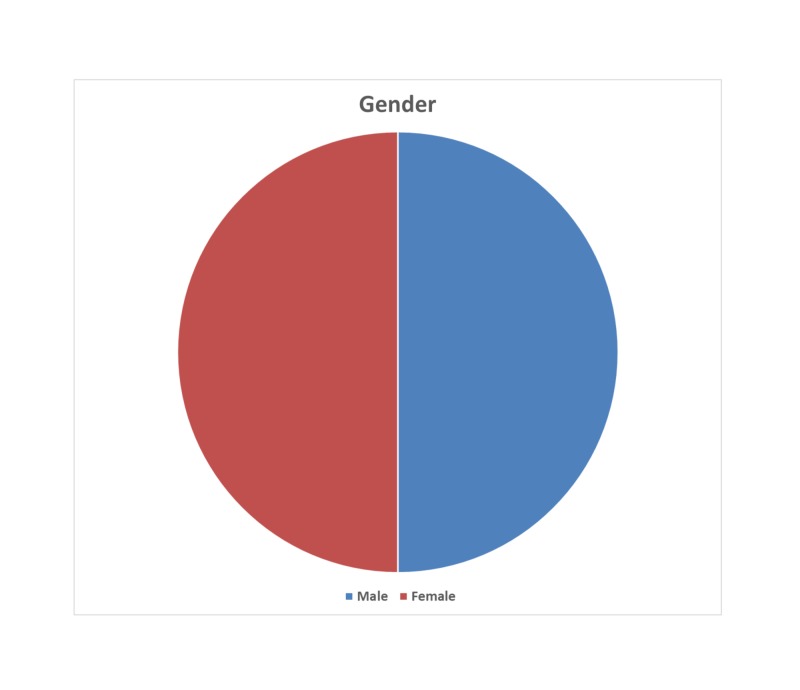
Gender distribution of the patients

**Figure 2:**
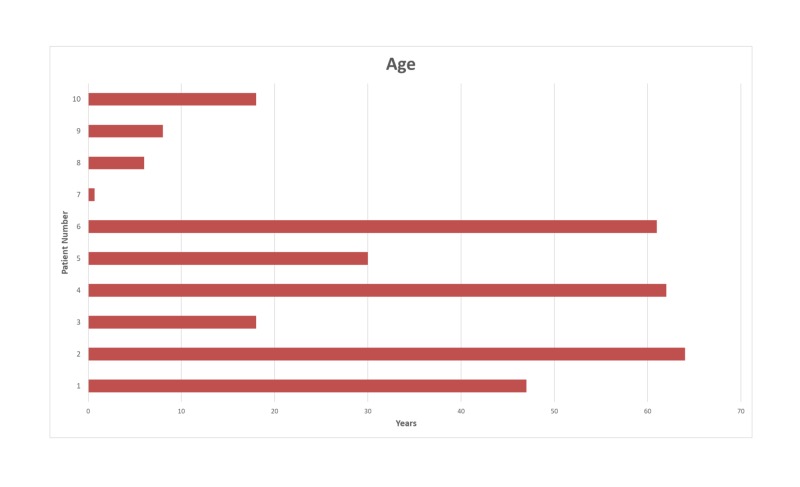
Age distribution of the patients

We performed a retrospective analysis of neurophysiological monitoring data from 10 consecutive spinal surgeries. The data were collected from two medical centers in two countries. The patients consisted of five women and five men with ages ranging from eight months to 67 years (median age: 49 years) (Figures 1-2). Seven patients were diagnosed with intradural tumors, and three patients with tethered cord syndrome. Our neuromonitoring protocol included bilateral upper and lower limbs SSEP, TCeMEPs, spontaneous EMG (s-EMG) and t-EMG from the EAS, EUS, and lower extremity muscles. For s-EMG, t-EMG, and TCeMEPs recording from the EUS muscles, a urinary catheter with an embedded bipolar urethral electrode (Signal Gear, Prosperity, SC, USA) was used [3]. A train of four (TOF) test was also performed from the abductor hallucis muscles in the foot.

Anesthesia protocol

All the procedures were performed under general anesthesia utilizing total intravenous anesthesia (TIVA) with propofol and remifentanil infusions. In all patients, a short-acting neuromuscular blocking agent was used for intubation. A train of four (TOF) was recorded from the abductor hallucis muscle for monitoring the level of muscle relaxant throughout the surgical procedure [8].

Intraoperative neurophysiological monitoring

A multimodality IONM protocol, including SSEP, TCeMEP, EMG, and TOF, was utilized for all patients. Standard recommended parameters were used for SSEP stimulation and recordings [9]. EMG and TCMEP responses were recorded from adductor brevis, vastus medialis, tibialis anterior, gastrocnemius, abductor hallucis, EAS, and EUS muscles. For evaluation of the pelvic floor and pudendal nerve monitoring, EAS and EUS muscles were added to the neuromonitoring protocol. For TCeMEP stimulation, corkscrew electrodes were placed on the patient’s scalp at C1, C2 and/or C3, C4 for. A train of 5-7 pulses was utilized (50-75 µsec duration) with a stimulation intensity range of 120 to 380 volts.

**Figure 3:**
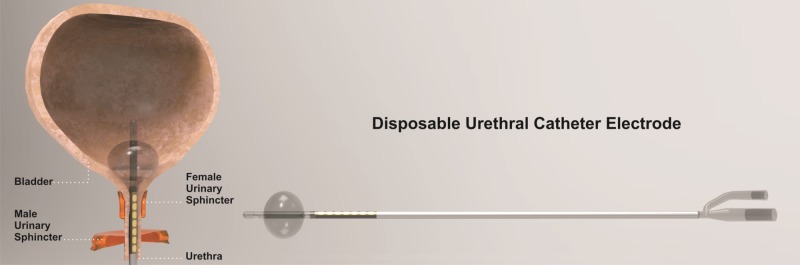
Urethral catheter A disposable Foley catheter with an embedded bipolar urethral catheter electrode contacts for electromyography (EMG) and transcranial electrical motor evoked potential (TCeMEP) recordings (used with permission from Signal Gear, Prosperity, SC, USA).

A urinary catheter with attached urethral electrodes was used for recording t-EMG, s-EMG, and TCeMEPs from the skeletal muscle of the external urethral sphincters [3] (Figure 3). The size of urethral catheter electrodes was on the French scale (Fr.). The diameter of each unit is approximately 0.33 mm (e.g., a 12 Fr catheter has a diameter of 4 mm). Urethral catheter electrode sizes used for children: 8 Fr. or 10 Fr. and for adults 14 Fr. or 16 Fr. An appropriate size of the urethral catheter electrode was used per the patient’s age, sex, urethral size, and weight.

A 32-channel Cadwell Cascade Pro (Cadwell Industries Inc, Kennewick, WA, USA) and Medtronic NIM-Eclipse (Medtronic, Inc., Minneapolis, MN, USA) were used for recorded multimodality IONM data during these surgical procedures.

## Results

**Figure 4:**
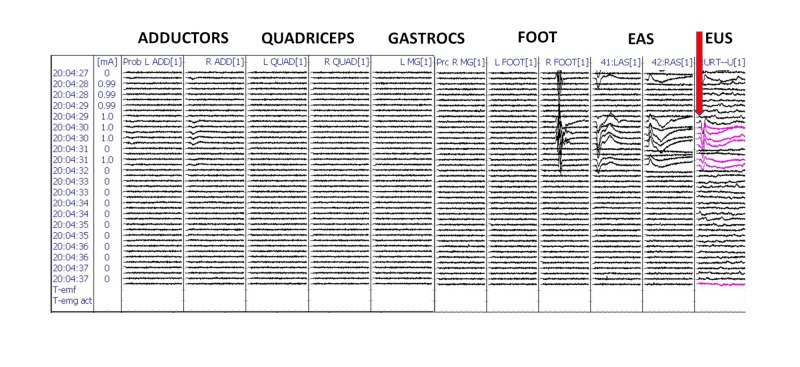
Triggered electromyography (t-EMG) recordings from the urethral sphincter electrodes in patient #1 Red arrow: urethral electrode compound muscle action potentials (CMAP) responses from the external urethral sphincter (EUS).

**Figure 5:**
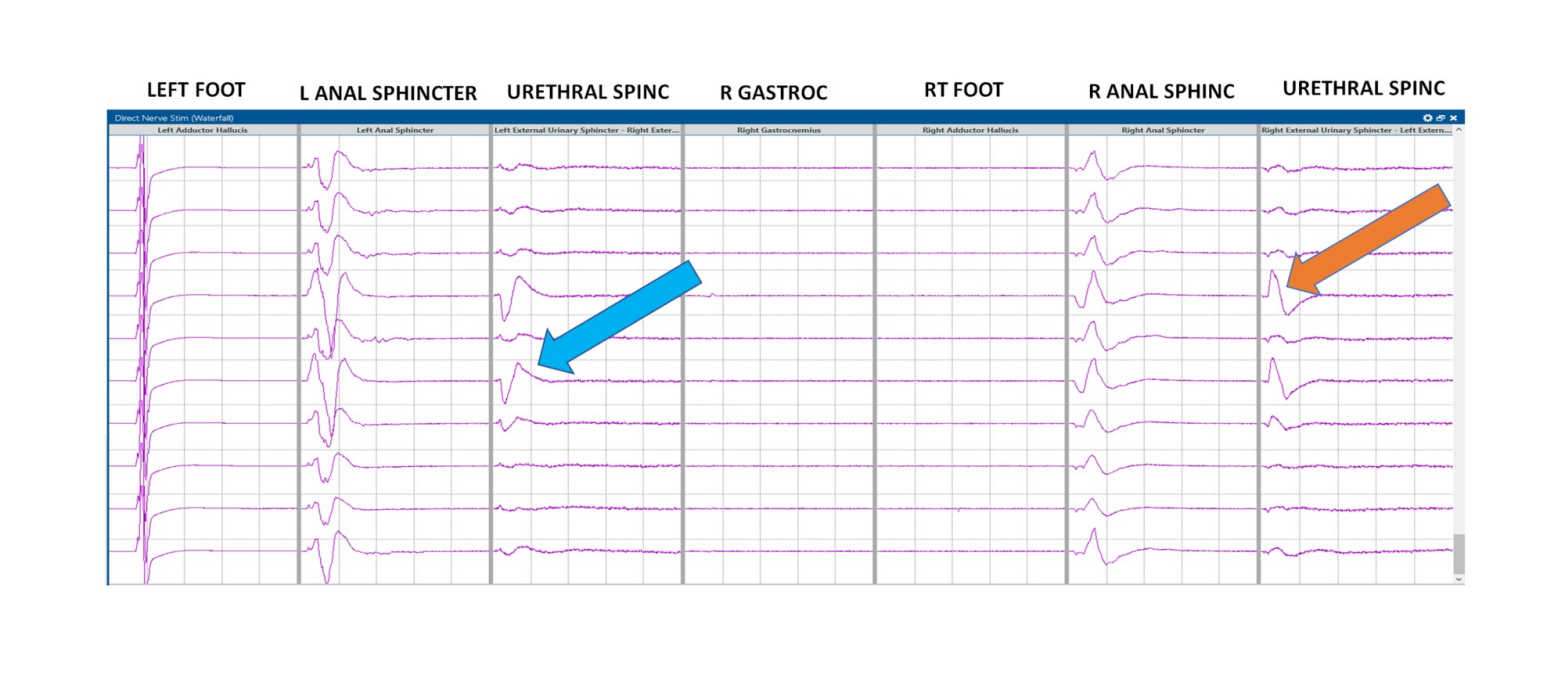
Triggered electromyography (t-EMG) recordings from the urethral sphincter electrodes in patient #9 Blue and orange arrows: compound muscle action potentials (CMAP) responses from the external urethral sphincter (EUS).

**Figure 6:**
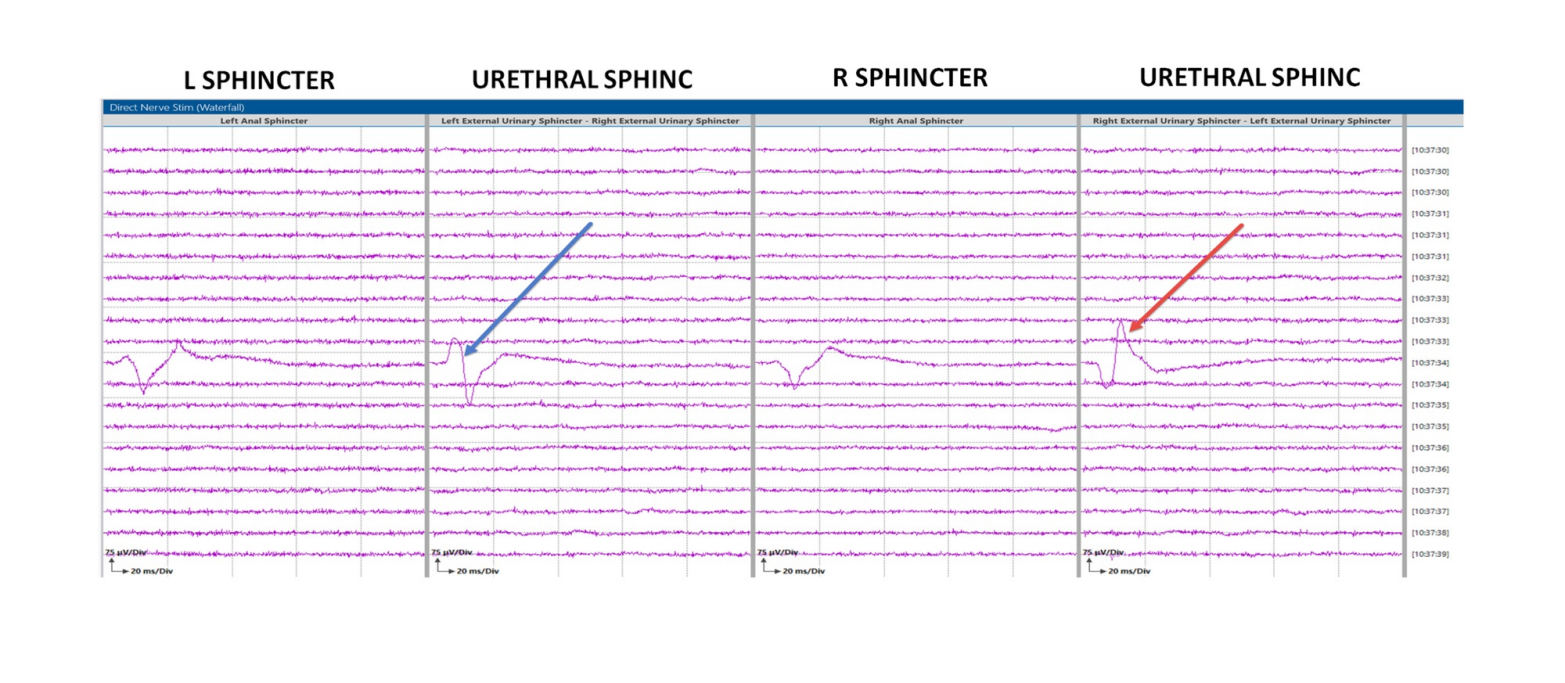
Triggered electromyography (t-EMG) data External urethral electrode compound muscle action potential (CMAP) responses in patient #2, indicated by the blue and red arrow.

We were able to successfully record t-EMG responses in all patients (100%) from the external urethral sphincter muscles (Figure 4). The morphology of the t-EMG responses from EUS was reliable and reproducible (Figures 5-6).

Only one patient presented preoperatively with bladder incontinence, urgency, and frequency. The patient’s frequency and urgency improved immediately in the postoperative phase, and the bladder function was normalized within two weeks of having the tumor removed. There were no false-negative or false-positive data in this study (Table 1).

**Table 1:** Triggered electromyography (t-EMG) responses recorded from the external urethral sphincter muscles (EUSM) M = male, F = female, P = present, Fr = Frank size, y = years, m = months, T-EMG = Triggered EMG.

Patient	Age	Sex	Diagnosis	Surgery	Catheter Size	T-EMG
1	47y	M	Cauda equina tumor	Laminectomy for tumor resection	16 Fr	P
2	64y	M	Conus tumor	Conus tumor resection	16 Fr	P
3	18y	F	Conus tumor	Conus tumor resection	14 Fr	P
4	62y	M	Conus tumor	Conus tumor resection	16 Fr	P
5	30y	M	Cauda equina tumor	Conus tumor resection	14 Fr	P
6	61y	M	Intradural tumor, L2-4	Laminectomy for tumor resection	16 Fr	P
7	8m	F	Tethered cord	Tethered cord release	8 Fr	P
8	6y	F	Tethered cord	Tethered cord release	10 Fr	P
9	8y	F	Tethered cord	Tethered cord release	10 Fr	P
10	18y	F	Tethered cord	Tethered cord release	16 Fr	P

## Discussion

Incontinence is a poor outcome that surgeons want to avoid when the risk factor for incontinence is present [10-11]. Using t-EMG with UES muscle recordings provides a novel way the neurophysiologist and surgeon may be able to detect impending neurological sequelae, where other modalities that are currently used for neuromonitoring purposes have been focused on spinal cord monitoring [12].

For patients with an impaired bladder function due to an incomplete spinal cord injury (SCI) as a complication, we discovered that the pudendal SSEP’s do not represent the autonomic function of the bladder, but rather somatic nerve fibers of the EUS showing that reproducible pudendal SSEP’s are indicative of recovery of bladder function [13]. Another study that looked at EUS function on patients with SCI measured the level of recovered bladder function six months post trauma broken down into normal, impaired, and absent. Urodynamic examinations were done by using two micro transducer catheters to read urethral and rectal pressures as well as SSEP recordings from the extremities and gentiles [14]. They found that in acute tetraplegia patients, 27% recovered to normal bladder function, with 73% developing an upper motor neuron (UMN) lesion. The voluntary EUS function was normal in 27%, impaired in 39%, and absent in 34%. Regarding paraplegic patients, only 10% recovered normal bladder function while 47% developed a UMN lesion, and 33% developed a lower motor neuron (LMN) lesion while 8% had a combined lesion. The EUS was normal in the 10%, impaired in 30% and absent in 60% [14].

## Conclusions

In this series, we were able to acquire t-EMG in 100% of patients when recorded from the EUS using a urethral catheter with electrodes built into it. T-EMGs can be attempted in surgeries that put the function of the pelvic floor at risk. More studies are needed to establish better statistical methods, better modality efficacy, and a better understanding of intraoperative countermeasures that may be employed when an alert is encountered to prevent impending neurological deficits. Currently, there are no studies published about neurophysiological monitoring of the urinary bladder or EUS function by utilizing t-EMG. This can be very helpful in surgeries involving the nerve roots supplying the EUS. This is the first study, to our knowledge, that shows a possibility for monitoring motor evoked potentials with EUS recordings in addition to the regularly monitored EAS.
